# Vibration-Assisted Roll-Type Polishing System Based on Compliant Micro-Motion Stage

**DOI:** 10.3390/mi9100499

**Published:** 2018-09-29

**Authors:** Yan Gu, Xiuyuan Chen, Jieqiong Lin, Mingming Lu, Faxiang Lu, Zheming Zhang, Hao Yang

**Affiliations:** 1 School of Mechatronic Engineering, Changchun University of Technology, Changchun 130012, China; chenxiuyuan1994@126.com (X.C.); lumm@ccut.edu.cn (M.L.); lufxxer@163.com (F.L.); 15543681350@163.com (Z.Z.); 2 Changchun Equipment and Technology Research Institute, Norinco Group, Changchun 130012, China; H.yang@126.com

**Keywords:** vibration-assisted, roll-type polishing system, micro-motion stage, grey wolves optimization (GWO) algorithm, silicon carbide (SiC) ceramic

## Abstract

This paper aims to create a high-quality surface based on the linear contact material removal mechanism. For this paper, a piezo-driven, flexure-based micro-motion stage was developed for the vibration-assisted roll-type precision polishing system. Meanwhile, the compliance matrix method was employed to establish the amplification ratio and compliance model of the flexure mechanism. The dimensions of the mechanism were optimized using the grey wolves optimization (GWO) algorithm, aiming to maximize the natural frequencies. Using the optimal parameters, the established models for the mechanical performance evaluation of the flexure stage were verified with the finite-element method. Through closed-loop test, it was proven that the proposed micro-motion stage performs well in positioning micro motions. Finally, high quality surface using silicon carbide (SiC) ceramic with 36 nm Sa was generated by the independently developed vibration-assisted roll-type polishing machine to validate the performance of the established polishing system.

## 1. Introduction

Advanced ceramic materials have been found to be used in a new generation of space-to-ground optical information collection systems, so as to make the optical systems constantly have thermal stability and a high stiffness to weight ratio. The growth of optical camera key components that are made of a high-quality optical reflector is also encouraged [1]. Silicon carbide (SiC) ceramic is a competitive representative material for establishing the ideal space for reflecting mirrors by the advantages of chemical inertness and corrosion resistance. However, there also remains difficulties with SiC linked with the inherent properties of high brittleness and low fracture toughness [2,3]. Among the various machining methods for SiC ceramic materials, polishing stands out as the most suitable way to produce ultra-precision surfaces. In particular, polishing is key in aeronautics for better mechanical performance [4,5].

Traditionally, the computer numerical control (CNC) polishing method is widely adopted for processing high-cost, aspheric optics. There is a positive correlation between the accuracy and duration of polishing. To remove contour errors, Becker et al. designed a CNC polisher with a coordinate measuring machine (CMM), which feeds error data draw back to the polisher and determines a proper polishing duration [6]. Inspired by uncertainty processes, Jones et al. developed a large CNC aspheric optics polishing machine that polished the target optical profile alternatively by interferometric inspection method [7]. Mori et al. extended the theory on an atomic scale fracture to CNC shaping and finishing of semiconductors, creating a polishing tool that applied a constant load from the normal direction on the substrate surface; in the meantime, loose abrasives were supplied. Unlike the conventional CNC polishing method, this tool wrapped up the rotating sphere with a relatively soft polyurethane material [8,9]. Though diamond polishing is still the most widely used finishing process, some subsurface damages and cracks may also be generated by the great force and high temperature. In particular, the rapid blunting and wear of the polishing tool will shorten the service life, suppress the productivity, and push up the cost. To solve these defects, many studies have been done to apprehend the polishing mechanism. For example, vibration assistance has been introduced to enhance the surface integrity and machining efficiency of hard and brittle materials through the alteration of process kinematics. Suzuki et al. applied ultrasonic vibration transversely to the workpiece surface under constant pressure, using loose abrasive and a micro-spherical polyurethane tool. In the subsequent study, an ultrasonic two-axis vibration-assisted polishing machine with hybrid piezo-electric actuators was developed for finishing micro-aspheric optics molds with ultra-precise high numerical aperture (NA) [10,11]. Both non-rotating tools were applicable to small aspherical targets, which were based on the measured material removal depth and the surface morphologies. Zhao et al. conducted ultrasonic vibration-assisted polishing (UVAP) of cylindrical groove arrays on silicon carbide (SiC), revealing that the two-body abrasion mechanism could be transferred to the three-body abrasion mechanism at low relatively speed and polishing force [12]. To improve the machining efficiency, elliptical ultrasonic-assisted grinding (EUAG) has been proposed by Liang et al. and successfully applied to the machining of mono-crystal sapphire, and carried out the brittle-ductile transition characteristics using single diamond abrasive grain [13]. Through 2D ultrasonic assisted polishing, Yu et al. enhanced the material removal rate (MRR) and reduced the surface roughness of Inconel 718 nickel-based alloy [14].

Despite the aforementioned efforts, most vibration-assisted polishing devices are still resonant types that convert electrical energy to mechanical energy using the piezo-electric transducers. Some of them even resort to ultrasonic frequencies (>20 kHz). The resonant types are more energy efficient, but they can only work at certain discrete frequencies. Comparatively speaking, non-resonant types typically use the piezo-driven flexure mechanism and can operate at a large range of continuous frequencies. Considering the complexity of the polishing process, it is highly necessary to design non-resonant, multi-dimensional vibration-assisted polishing device. So many researchers have reported numerous research results on non-resonant, multi-dimensional devices in recent years [15,16]. Below is a brief review of related studies. Suzuki et al. proposed a low contact force polishing system using 2D low frequency vibrations (2DLFV) based on the piezo-driven flexure mechanism, which was capable of deterministic surface error correction of the complicated shapes on micro molds, as a result of its flexibility in view of applicable polishing tools [17]. Micromilling is in direct competition with laser and other mechanical surface treatments [18]. In order to generate specific surface textures and design hydrophobicity surface property, Chen et al. developed a method of the surface texture formation using non-resonant vibration assisted micro milling [19]. Gu et al. developed a non-resonant vibration-assisted polishing device (VAPD) with parallel-driven piezo-electric (PZT) actuators aiming to solve the processing limitations of hard and brittle materials, and the maximum working bandwidth could reach up to 1879 Hz [20]. Nevertheless, the new electronics manufacturing process is suitable for continuous production lines, but not compatible with the conventional batch-type polishing process. To overcome the limitations of the traditional polishing system, the roll-type linear chemical mechanical polishing (roll-CMP) system came into being, which relied on a low-friction removal material system for linear contact to reduce the flexural deformation of ultrathin substrates or brittle fracture of large rectangular glass substrates [21]. Targeting the material removal system, Lee et al. investigated the influences of the process parameters on the mean MRR (MRRavg) and non-uniformity (NU) in a roll-CMP system and proposed a mathematical model for linear roll-CMP to disclose the effects of polishing pads on the MRR of Cu, which laid a solid theoretical basis for the roll-CMP process [22,23].

Motivated by the above-mentioned problems, we target the development of an elliptical vibration-assisted roll-type polishing (EARP) system considering the flexural mechanism to extend the capabilities of existing mechanisms for precision polishing. This system has the following advantages: it works in the non-resonant mode and can deliver large working stroke; benefiting from the principle of linear contact, ultrathin substrates without brittle fracture could be realized; the flexible system supports frequency adjustment and suits the continuous production line in new electronics production; high polishing accuracy can be realized through the integration of feed and machining modules.

## 2. Structural Design and Prototype Development

### 2.1. Assembly of the EARP System

The EARP system consists of a ball-screw feed drive system, a polishing roller processing system, and an XY micro-motion stage with a large workspace. In particular, the ball-screw feed drive system should be noted, which includes servo motors, ball-screws, couplings, supporting bearings, linear guides, and other machine structures. Another part is the polishing roller processing system, which involves an aluminum roller, a hard polyurethane polishing pad (to ensure the flatness of substrate surface), and a DC motor. The polishing roller processing system could achieve its own rotation and translation in the XZ plane. The resolution of motion of the ball-screw feed drive system is 1 μm in the *Z* direction. The value of the roller scanning and rotation speed is calculated based on the requirement of the workpiece surface quality and the NC program is generated by the personal computer. The XY micro-motion stage is driven by two PZT actuators (PZT1 and PZT2), owing to its high input stiffness. Considering that fixing holes can be easily machined with a tolerance of ±5 µm, precise assembly of the components is not impossible nowadays.

**Figure 1:**
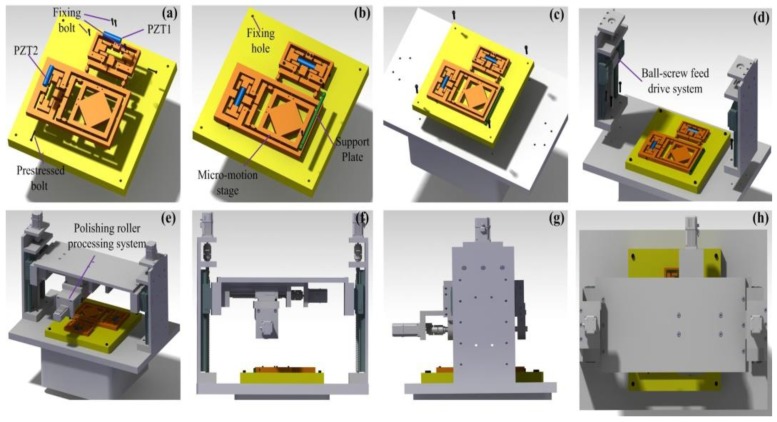
Elliptical vibration-assisted roll-type polishing (EARP) assembly procedure. (**a**–**c**) components assembly; (**d**,**e**) base mounting; (**f**–**h**) front, side, and top views. PZT—piezo-electric.

The computer-aided design assembly processes of the EARP system are shown graphically in Figure 1. The components of the stage can be assembled together through two steps (Figure 1a,b). The fixing bolts in Figure 1a are the fixed holes constrained in all directions, and the support plate in Figure 1b is only limited by the contact area in the X direction. In the additional steps shown in Figure 1c–e, the micro-motion stage, the ball-screw feed drive system, and the polishing roller processing system are assembled together and fixed at the base. The front, side, and top views of the assembled model are shown in Figure 1f–h.

### 2.2. Vibration Trajectory of the XY Micro-Motion Stage

As reviewed in the introduction, some parameters can be arbitrarily altered by the non-resonant vibration device. It is simple and directly generates the ellipse with two orthogonal actuators, compared with those devices with two parallel actuators. The micro-motion stage aims to vibrate at a certain frequency and time about the center point O in the plane XOY. According to the basic vibration principles, the vibration process of the 2D micro-motion stage with variable parameters can be expressed as

(1) $$\begin{array}{l} x=A_{x}\sin\left(2\pi f_{1}t\right)\\ y=(1-\eta^{\prime })\cdot A_{y}\sin\left(2\pi f_{2}t+\varphi \right) \end{array}$$

where *A_x_* and *A_y_* are the signal amplitudes in the *x* and *y* directions, respectively; *f*_1_ and *f*_2_ are the signals frequencies in the directions of *x* and *y*, respectively; φ is the phase difference between the initial phases in *x* and *y* directions; and $\eta^{\prime }$ is the displacement loss rate induced by hinges 7, 8, 9, and 10 in the *y* direction. Then, the stage trajectories were analyzed with different vibration parameters.

**Figure 2:**
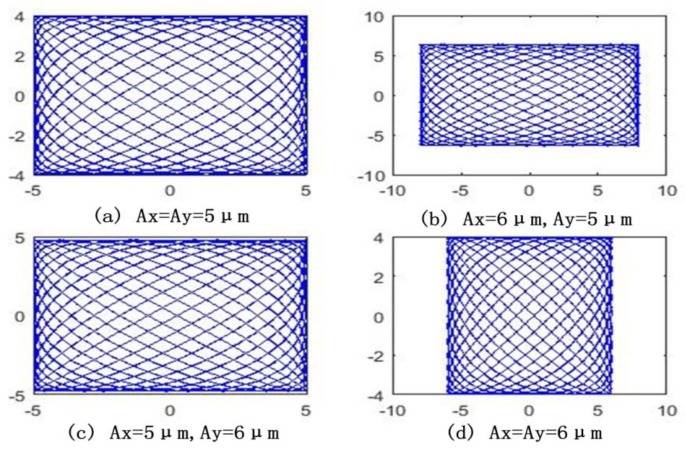
The composition trajectory for different amplitudes. (**a**) same amplitudes: *A_x_* = *A_y_* = 5 μm; (**b**) different amplitudes: *A_x_* = 6 μm, *A_y_* = 5 μm; (**c**) different amplitudes: *A_x_* = 5 μm, *A_y_* = 6 μm; (**d**) same amplitudes: *A_x_* = *A_y_* = 6 μm.

The trajectory of the stage in the XOY plane exhibited as a Lissajous curve when signals with different frequencies were applied to directions of *x* and *y*. The parameters of *f*_1_ = *f*_2_, φ=π/2 are provided in Equation (1). Figure 2 shows the effects of 2D vibration amplitudes on the closed trajectory motion of the stage.

As shown in Figure 2, the vibration amplitudes in the *x* and *y* directions formed a square trajectory area under the same conditions. The magnitude of amplitudes affects only the size for the area, without affecting the uniformity of the Lissajous figures.

### 2.3. Design of the EARP System

**Figure 3:**
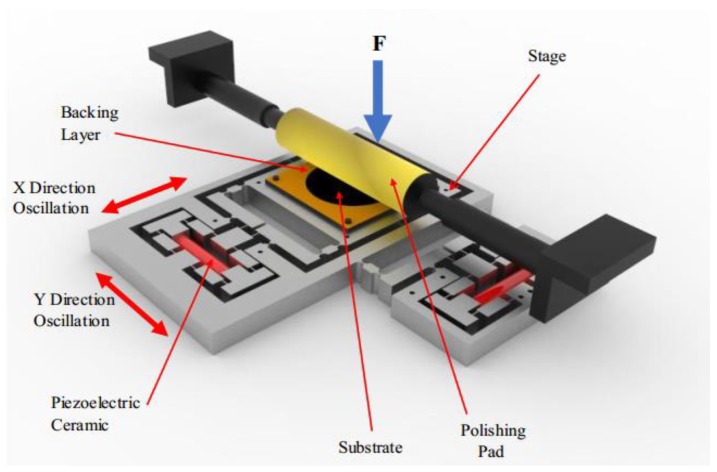
The schematic diagram of elliptical vibration-assisted roll-type polishing (EARP) system.

The EARP system is designed with a XY micro-motion stage, a roller, and a pair of PZT actuators, as shown in Figure 3. The substrate should be placed on the stage and attached to the rubber backing layer. The polishing pad is tightly coiled around the high-stiffness roller, which can dampen the vibrations at a high rolling speed.

**Figure 4:**
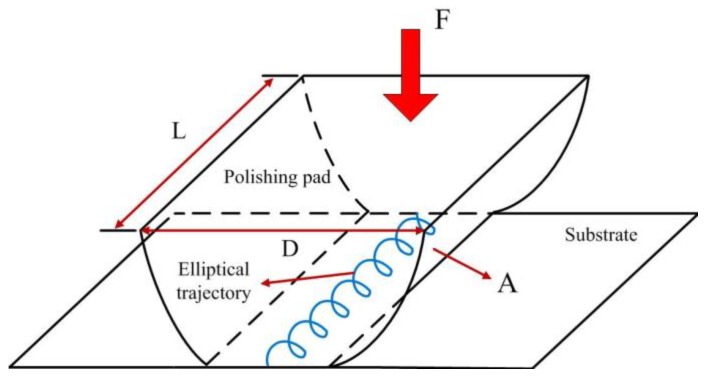
Schematic of contact area in the EARP system.

As the conventional roll-type polishing system performs polishing with line-contact material removal mechanism, the polishing contact area is much smaller than that of the rotary CMP system. Accordingly, the abrasives directly involved in the polishing are insufficient, which impacts the surface roughness. In order to raise the amount of abrasives involved in polishing, an elliptical vibration is introduced in the processing, as shown in Figure 4. *L* is the roller length, *D* is the roller diameter, and *A* is the apparent area of contact between the pad and the substrate.

## 3. Determination of Amplification Ratio and Compliance Matrix Modeling

### 3.1. Determination of Amplification Ratio and Compliance Matrix Modelling

**Figure 5:**
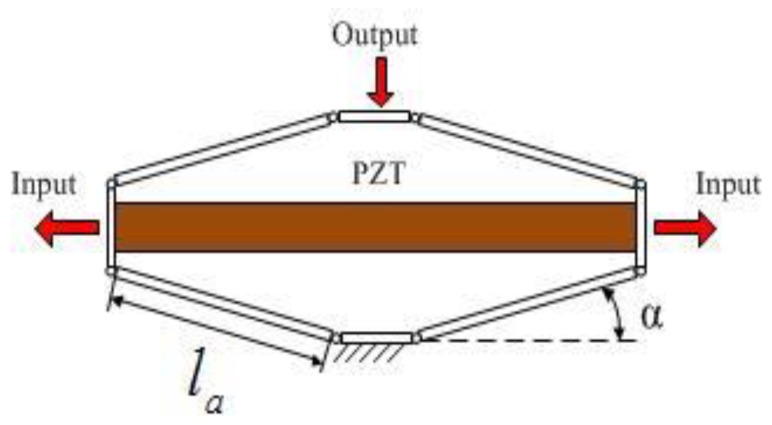
Ideal model of bridge-type mechanism.

Modelling may be the suitable way to improve the use of the EARP on several applications [24]. Before the kinematics analysis, it is necessary to simplify the bridge-type mechanism as an ideal multi-rigid body mechanism with ideal pivots, as shown in Figure 5. Let *l_a_* be the length of the arm and *α* be the angle between the connecting line of arm pivots and the horizontal line.

**Figure 6:**
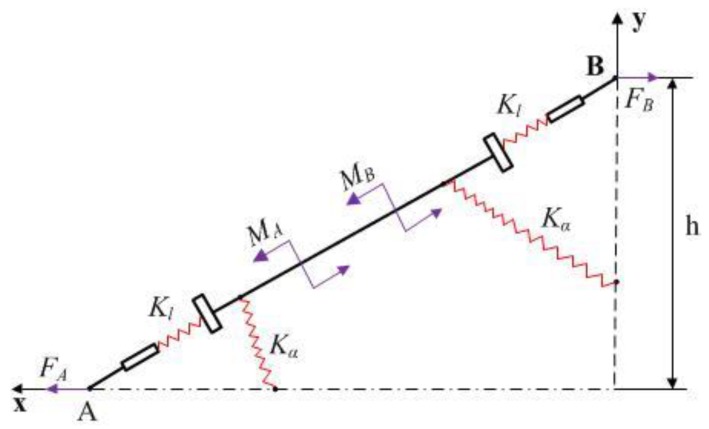
Quarter mechanic model of bridge-type flexure hinge.

Considering the huge topological differences, each arm in the bridge-type mechanism is simulated as a link between two flexure hinges, and each flexure hinge is modelled as an elastic beam. As the structure is symmetrical, only one bridge arm of the mechanical model needs to be analyzed. Through kinematic analysis, the static mechanics model of the flexure hinge AB can be simplified as a quarter mechanic model, as shown in Figure 6.

**Figure 7:**
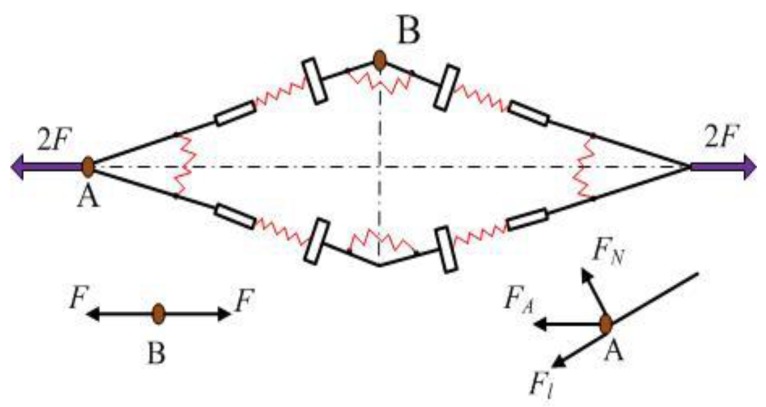
The mechanics of bridge-type flexure hinge.

As shown in Figure 7, there are only two symmetric, horizontally balanced forces at point B. Meanwhile, the arm AB has two moments, and the force at point A is provided by point B. From the force equilibrium theory, the equations showing that $F_{A}=F_{B}=F$ and $2M_{A}=2M_{B}=M$ can be easily derived.

The equation would appear when the force *F* is applied at point A (Figure 7) [25].

(2) $$\frac{M}{F_{l}}=\frac{Fh}{F\cos\alpha }=\frac{2K_{\alpha }\Delta \alpha }{K_{l}\Delta l}$$

At point A on arm AB,

(3) $$F\Delta x=F_{l}\cdot \Delta l+M\cdot \Delta \alpha =F\cos\alpha \cdot \Delta l+Fh\cdot \Delta \alpha$$

The bridge-type mechanism often has an extremely small deformation, that is, the Δα is very small. Therefore, the chord length produced by the rigid body rotation is approximately equal to the arc length corresponding to Δα [26]. Then, Δy can be expressed as follows:

(4) $$\Delta y=l_{a}\cos\alpha \cdot \Delta \alpha ,$$

**Figure 8:**
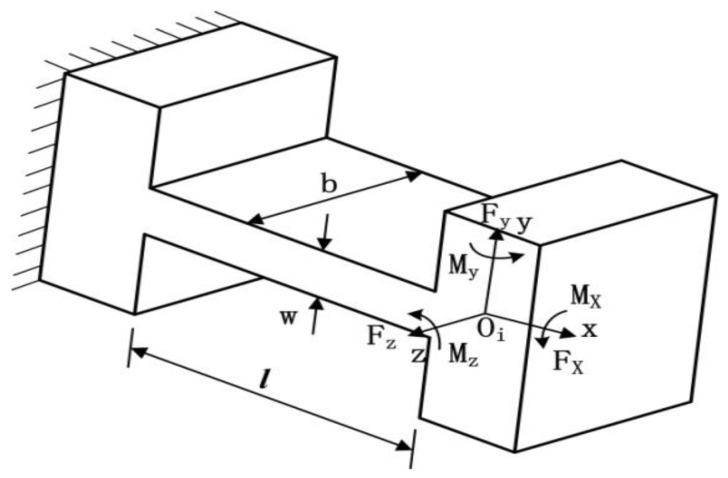
Coordinate system of right-angle flexure hinge.

According to beam theory, the compliance matrix of the right-angle hinge can be obtained by the following [27]:

(5) $$C^{h}=\left[\begin{array}{ccc} \frac{l}{Ebw} & 0 & 0\\ 0 & \frac{4l^{3}}{3Ebw^{3}}+\frac{l}{Gbw} & \frac{6l^{2}}{Ebw^{3}}\\ 0 & \frac{6l^{2}}{Ebw^{3}} & \frac{12l}{Ebw^{3}} \end{array}\right]$$

where *E* is the elastic modulus and *G* is the shear modulus of the hinge material. The coordinate system of the right-angle hinge is shown in Figure 8.

According to Equation (5), $K_{l}$ and $K_{\alpha }$ can be derived as follows:

(6) $$K_{l}=\frac{Ebw}{l},\;K_{\alpha }=\frac{Ebw^{3}}{12l}$$

Considering the elastic stiffness of the right-angle hinge, the theoretic displacement amplification ratio can be calculated as

(7) $$Ramp_{1}=\frac{\Delta y}{\Delta x}=\frac{l_{a}\cos\alpha }{\frac{\cos^{2}\alpha \cdot w^{2}}{6h}}+h$$

The ideal displacement amplification ratio of the lever model is

(8) $$Ramp_{2}=\frac{L}{m}$$

where *L* is the total length of the lever and *m* is input length.

Thus, the actual amplification ratio of the displacement amplifier can be obtained as

(9) $$Ramp=Ramp_{1}\cdot Ramp_{2}=\{\begin{array}{ll} \frac{l_{a}\cos\alpha \cdot L}{(\frac{\cos^{2}\alpha \cdot \omega^{2}}{6h}+h)m} & x\;direction\\ \left(1-\eta^{\prime }\right)\cdot \frac{l_{a}\cos\alpha \cdot L}{(\frac{\cos^{2}\alpha \cdot \omega^{2}}{6h}+h)m} & y\;direction \end{array}$$

### 3.2. Compliance Matrix Modelling of XY Micro-Motion Stage

#### 3.2.1. Compliance Matrix Method

According to the simplified model of Wu & Zhou [28], the compliance matrix of the right-circular hinge in its local coordinate system can be defined as follows:

(10) $$C^{f}=\left(\begin{array}{ccc} c_{11} & 0 & 0\\ 0 & c_{22} & c_{23}\\ 0 & c_{32} & c_{33} \end{array}\right)$$

**Figure 9:**
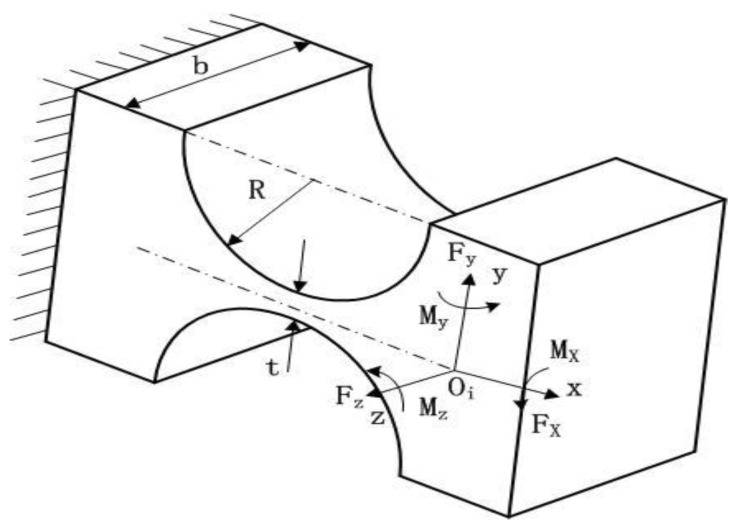
Coordinate system of right-circular hinge.

The coordinate system of the right-circular hinge is shown in Figure 9.

As the links have much higher stiffness than flexure hinges, the motions of micro-motion stage can be viewed as the elastic deformation of the flexure hinges [29]. According to Figure 8 and Figure 9, when a load vector $\bar{F}={\left[\begin{array}{ccc} f_{x} & f_{y} & m_{z} \end{array}\right]}^{T}$ is applied on point $O_{i}$ of the flexure in/around certain axes, the displacement $\bar{D}={\left[\begin{array}{ccc} d_{x} & d_{y} & \theta_{z} \end{array}\right]}^{T}$ of that point in its local coordinate system $O_{i}-xy$ can be described as

(11) $$\bar{D}=C_{Oi}\bar{F}$$

where $C_{Oi}$ is the compliance matrix of the flexure hinge in its local coordinate system $O_{i}-xy$ [30]. Note that the compliance factors in the matrices of the right-angle hinge and the right-circular hinge can be derived from Equations (5) and (10), respectively.

For a flexure hinge, the compliance matrix can be transferred from the local coordinate system $O_{i}-xy$ to another coordinate system $O_{j}-xy$ by the following equation

(12) $$C_{Oj}=T_{i}^{j}C_{Oi}{\left(T_{i}^{j}\right)}^{T}$$

where the transformation matrix $T_{i}^{j}$ can be described,

(13) $$T_{i}^{j}={\bar{R}}_{i}^{j}\cdot {\bar{P}}_{i}^{j}$$

The matrix $P_{i}^{j}$ can be obtained by

(14) $${\bar{P}}_{i}^{j}=\left[\begin{array}{ccc} 1 & 0 & r_{y}\\ 0 & 1 & -r_{x}\\ 0 & 0 & 1 \end{array}\right]$$

If the matrix ${\bar{R}}_{i}^{j}$ rotates around the *x*, *y* and *z* axes, it can be written as

(15) $${\bar{R}}_{x}^{j}=\left[\begin{array}{ccc} 1 & 0 & 0\\ 0 & \cos\alpha & \sin\alpha \\ 0 & -\sin\alpha & \cos\alpha \end{array}\right],\;{\bar{R}}_{y}^{j}=\left[\begin{array}{ccc} \cos\beta & 0 & -\sin\beta \\ 0 & 1 & 0\\ \sin\beta & 0 & \cos\beta \end{array}\right],\;{\bar{R}}_{z}^{j}=\left[\begin{array}{ccc} \cos\varepsilon & \sin\varepsilon & 0\\ -\sin\varepsilon & \cos\varepsilon & 0\\ 0 & 0 & 1 \end{array}\right]$$

where *α*, *β*, and *ε* are the rotation angles of *x*, *y*, and *z* axes, respectively.

#### 3.2.2. Output Compliance Modelling

**Figure 10:**
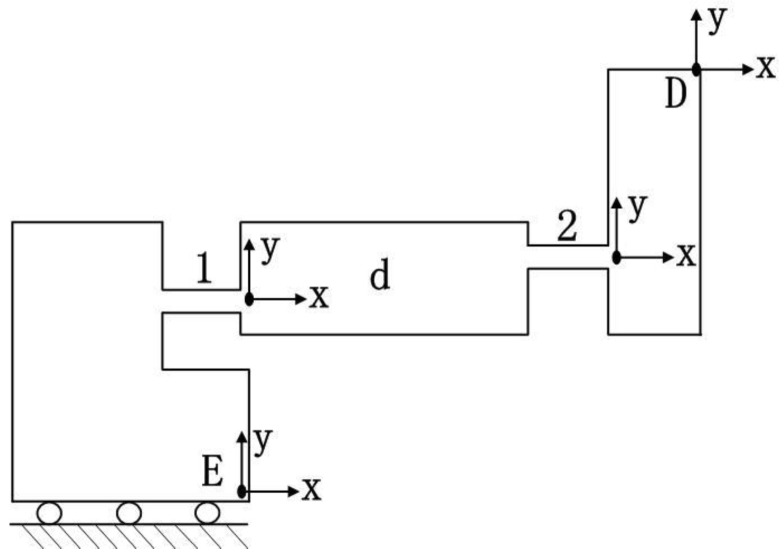
Quarter model of bridge-type mechanism.

Because of the double symmetric property, a quarter of the bridge-type mechanism is modelled (Figure 10). In the coordinate system *E*-*xy*, the compliance of flexure hinge *d* can be expressed as follows:

(16) $$C_{d}^{E}=C_{1}^{E}+C_{2}^{E}=T_{1}^{E}C_{1}{\left(T_{1}^{E}\right)}^{T}+T_{2}^{E}C_{2}{\left(T_{2}^{E}\right)}^{T}$$

where $C_{i}^{E}$ is defined as the compliance of flexure hinge *i* with respect to the point *E* in the coordinate system *E*-*xy*, $C_{i}$ is the compliance of flexure hinge *i* in the local coordinate system, and $T_{i}^{E}$ is the transformation matrix from the coordinate system *E*-*xy* to the local coordinate system.

The compliance of the left section can be written as

(17) $$C_{Left}^{E}=C_{d}^{E}+{\bar{R}}_{x}^{E}\left(\pi \right)C_{d}^{E}{\left({\bar{R}}_{x}^{E}\left(\pi \right)\right)}^{T}$$

**Figure 11:**
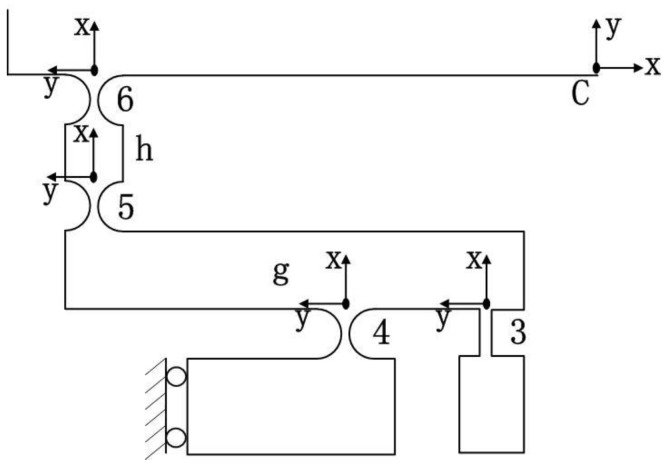
Half model of lever mechanism.

Thanks to the symmetric property, half of the lever mechanism is simulated (Figure 11). Then, the compliance of the left limb *M* in the coordinate system *C*-*xy* can be described as

(18) $$\begin{array}{c} C_{Left}^{C}={\left\{{\left[T_{E}^{C}C_{Left}^{E}{\left(T_{E}^{C}\right)}^{T}+T_{3}^{C}C_{3}{\left(T_{3}^{C}\right)}^{T}\right]}^{-1}+{\left[T_{4}^{C}C_{4}{\left(T_{4}^{C}\right)}^{T}\right]}^{-1}\right\}}^{-1}+T_{5}^{C}C_{5}{\left(T_{5}^{C}\right)}^{T}+T_{6}^{C}C_{6}{\left(T_{6}^{C}\right)}^{T} \end{array}$$

Hence, the compliance of limb *M* in the coordinate system *O*-*xy* can be obtained as

(19) $$C_{M}^{O}=T_{C}^{O}{\left\{{\left(C_{Left}^{C}\right)}^{-1}+{\left[{\bar{R}}_{y}^{C}\left(\pi \right)C_{Left}^{C}{\left({\bar{R}}_{y}^{C}\left(\pi \right)\right)}^{T}\right]}^{-1}\right\}}^{-1}{\left(T_{C}^{O}\right)}^{T}$$

Similarly, the compliance matrix of flexible limb *N* can be expressed as

(20) $$C_{N}=T_{O}^{N}C_{M}^{O}{\left(T_{O}^{N}\right)}^{T}$$

Therefore, the output compliance of the micro-motion stage in the coordinate system *O*-*xy* can be derived as

(21) $$\begin{array}{c} K_{O}={\left(C_{O}\right)}^{-1}={\left[T_{N}^{O}C_{N}{\left(T_{N}^{O}\right)}^{T}\right]}^{-1}+{\left[{\left(C_{7}^{O}\right)}^{-1}+{\left(C_{8}^{O}\right)}^{-1}+{\left(C_{9}^{O}\right)}^{-1}+{\left(C_{10}^{O}\right)}^{-1}\right]}^{-1}+{\left(C_{M}^{O}\right)}^{-1} \end{array}$$

#### 3.2.3. Input Compliance Modelling

**Figure 12:**
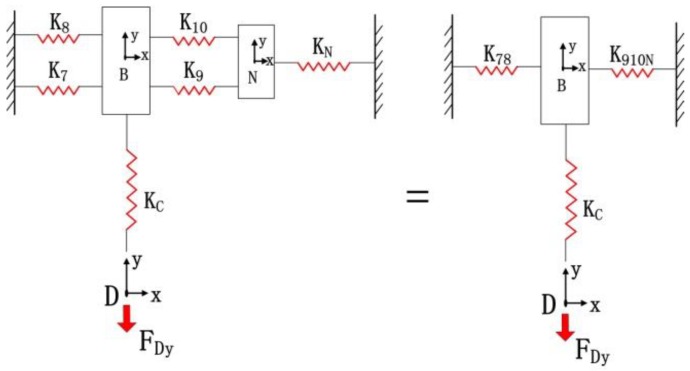
Stiffness model of the micro-motion stage in the *y*-direction with one limb actuated.

Excluding the bridge-type amplifier in limb *M*, the compliance model of the micro-motion stage is simulated as the stiffness model in Figure 12. The bridge-type amplifier will tolerate the force applied by the rest of the stage at the interface *D*. Then, integral compliance of chains 7 and 8 with respect to point *B* can be derived as

(22) $$C_{78}={\left[{\left(C_{7}^{B}\right)}^{-1}+{\left(C_{8}^{B}\right)}^{-1}\right]}^{-1}$$

The two hinges are connected to the limb *N* of chains 9 and 10; thus, we have

(23) $$C_{910N}=T_{N}^{B}C_{N}{\left(T_{N}^{B}\right)}^{T}+{\left[{\left(C_{9}^{B}\right)}^{-1}+{\left(C_{10}^{B}\right)}^{-1}\right]}^{-1}$$

Therefore, the compliance of point *D* can be calculated as

(24) $$C_{D}={\left[{\left(C_{78}^{D}\right)}^{-1}+{\left(C_{910N}^{D}\right)}^{-1}\right]}^{-1}+C_{C}^{D}$$

Let $C_{D}^{A}$ denote the compliance of point *D* with respect to input end *A* can be derived by

(25) $$C_{D}^{A}=T_{D}^{A}C_{D}{\left(T_{D}^{A}\right)}^{T}$$

**Figure 13:**
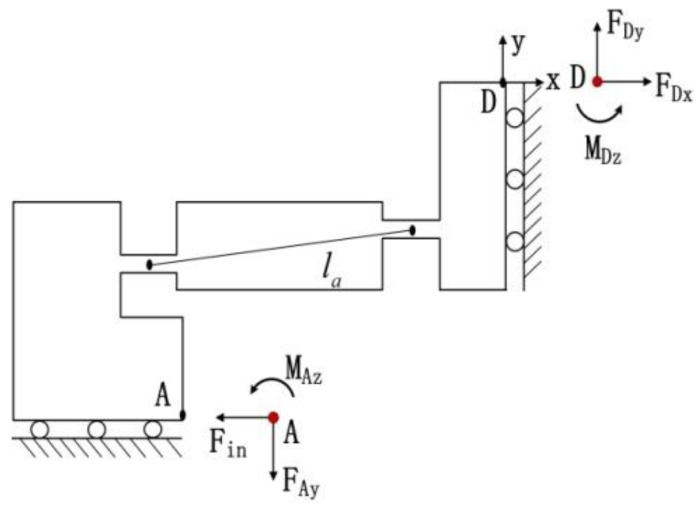
The free body diagram of quarter model of bridge-type mechanism.

Figure 13 shows the free body diagram of the quarter model of bridge-type mechanism. Assuming that point *D* remains fixed [31], the following equations can be obtained according to the force-deflection relationship at the input end *A*

(26) $$u_{Ay}=c_{22}F_{Dy}+c_{23}M_{Az}+c_{11}F_{in}$$

(27) $$\theta_{Az}=c_{32}F_{Dy}+c_{33}M_{Az}=0$$

where $c_{ij}$ (*i*, *j* = 1, 2 and 3) is the compliance factors in the *i*-th row and *j*-th column of the matrix $C_{D}^{A}$; and $F_{Dy}$ defines the force applied by the micro-motion stage excepting the bridge-type mechanism along the y direction, which is actuated by a force $F_{in}$ (input force of PZT).

Accordingly, the output displacement $u_{Ay}$ can be obtained as a function of input displacement $u_{in}$

(28) $$u_{Ay}=Ramp_{1}\cdot u_{in}$$

where $Ramp_{1}$ is the amplification ratio of the bridge-type model.

Then, the relationship between the load and deflection along the *y*-direction can be obtained as

(29) $$u_{Ay}=-d_{22}F_{Dy}$$

where $d_{22}$ is a compliance factor of matrix $C_{D}$.

According to Equations (26)–(29), the input compliance for the micro-motion stage in the *Y*-direction can be derived as follows

(30) $$k_{in}=\frac{F_{in}}{u_{in}}=\left(1+\frac{c_{22}}{d_{22}}-\frac{c_{23}\cdot c_{32}}{c_{33}\cdot d_{22}}\right)\cdot \frac{Ramp_{1}}{c_{11}}$$

**Figure 14:**
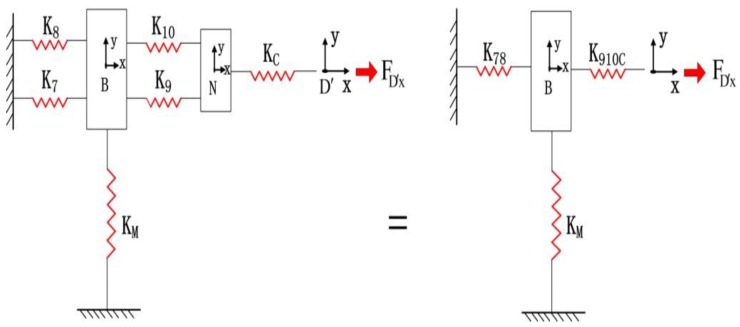
Stiffness model of the micro-motion stage in the *x*-direction with one limb actuated.

The stiffness model of the micro-motion stage with one limb actuated is established to calculate the input stiffness in the *x*-direction, as shown in Figure 14. The compliance of the point $D^{\prime }$ can be calculated by

(31) $$C_{D^{\prime }}={\left[{\left(C_{M}^{D^{\prime }}\right)}^{-1}+{\left(C_{78}^{D^{\prime }}\right)}^{-1}\right]}^{-1}+C_{910C^{\prime }}^{D^{\prime }}$$

The compliance of point $D^{\prime }$ with respect to input end $A^{\prime }$ is

(32) $$C_{D^{\prime }}^{A^{\prime }}=T_{D^{\prime }}^{A^{\prime }}C_{D^{\prime }}{\left(T_{D^{\prime }}^{A^{\prime }}\right)}^{T}$$

Then, the input compliance for the micro-motion stage in the *x*-direction can be derived as follows:

(33) $$k_{in}{}^{\prime }=\left(1+\frac{c_{22}{}^{\prime }}{d_{22}{}^{\prime }}-\frac{c_{23}{}^{\prime }\cdot c_{32}{}^{\prime }}{c_{33}{}^{\prime }\cdot d_{22}{}^{\prime }}\right)\cdot \frac{Ramp_{1}}{c_{11}{}^{\prime }}$$

where ${c^{\prime }}_{ij}$ (*i*, *j* = 1, 2 and 3) is the compliance factors in the *i*-th row and *j*-th column of matrix $C_{D^{\prime }}^{A^{\prime }}$, ${d^{\prime }}_{22}$ is a compliance factor of matrix $C_{D^{\prime }}$, and $Ramp_{1}$ is the amplification ratio of the bridge-type model. Machine stiffness could also be obtained in the experimental testing [32].

## 4. Dynamics Model

Compared with vector equations, energy methods can easily derive the energy equations of compliance mechanisms [33]. Thus, the natural frequencies of the 2D micro-motion stage are determined by energy equilibrium-based Lagrange equation, aiming to give a panorama of the free vibrations in the 2D micro-motion stage.

**Figure 15:**
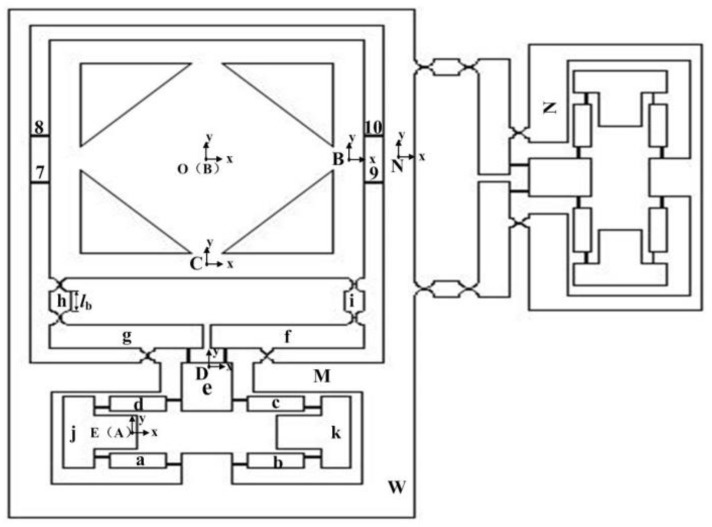
The 2-dimensional micro-motion stage.

The kinetic energies of the compliant system are expressed by the generalized coordinates, that is, the input-displacement variable $D_{I}=\left[\begin{array}{cc} d_{ix} & d_{iy} \end{array}\right]$. It is assumed that the kinetic energies come from the rigid links between the flexure hinges [34]. As shown in Figure 15, translational motions are generated by links *e*, *j*, and *k* in limb *M*; rotational motions are generated by links *g* and *f* in that limb; and both translational and rotational motions are generated by links *a*, *b*, *c*, *d*, *h*, and *i*. Moreover, the central point *O* of the motion stage moves along *x* and *y* axes without any rotation. The kinetic energy of limb *M*, consisting of the bridge-type mechanism and the lever model, can be expressed as

(34) $$\begin{array}{cc} T_{A}= & \frac{1}{2}m_{t}{\left(\frac{1}{2}{\dot{d}}_{iy}\right)}^{2}+\frac{1}{2}(\frac{1}{12})m_{t}l_{1}{}^{2}{\left(\frac{{\dot{d}}_{iy}}{l_{1}}\right)}^{2}+\frac{1}{2}m_{t}{\dot{d}}_{ix}{}^{2}+\frac{1}{2}m_{e}{\dot{d}}_{iy}{}^{2}+\frac{1}{2}m_{v}{\dot{d}}_{ix}{}^{2}+\frac{1}{2}m_{p}{\left(\frac{1}{2}{\dot{d}}_{ix}\right)}^{2}\\ & +\frac{1}{2}m_{p}{\dot{d}}_{iy}{}^{2}+\frac{1}{2}(\frac{1}{12})m_{p}l_{2}{}^{2}{\left(\frac{{\dot{d}}_{ix}}{l_{2}}\right)}^{2}+\frac{1}{2}(\frac{1}{12})m_{q}l_{3}{}^{2}{\left(\frac{{\dot{d}}_{iy}}{l_{3}}\right)}^{2} \end{array}$$

where

(35) $$m_{t}=m_{a}+m_{b}+m_{c}+m_{d}$$

(36) $$m_{v}=m_{j}+m_{k}$$

(37) $$m_{p}=m_{h}+m_{i}$$

(38) $$m_{q}=m_{g}+m_{f}$$

Because of the symmetric property, the kinetic energies of limbs *N* in the *x* direction can be considered as

(39) $$\begin{array}{cc} T_{B}= & \frac{1}{2}m_{t}{\left(\frac{1}{2}{\dot{d}}_{ix}\right)}^{2}+\frac{1}{2}(\frac{1}{12})m_{t}l_{1}{}^{2}{\left(\frac{{\dot{d}}_{ix}}{l_{1}}\right)}^{2}+\frac{1}{2}m_{t}{\dot{d}}_{iy}{}^{2}+\frac{1}{2}m_{e}{\dot{d}}_{ix}{}^{2}+\frac{1}{2}m_{v}{\dot{d}}_{iy}{}^{2}+\frac{1}{2}m_{p}{\left(\frac{1}{2}{\dot{d}}_{iy}\right)}^{2}\\ & +\frac{1}{2}m_{p}{\dot{d}}_{ix}{}^{2}+\frac{1}{2}(\frac{1}{12})m_{p}l_{2}{}^{2}{\left(\frac{{\dot{d}}_{iy}}{l_{2}}\right)}^{2}+\frac{1}{2}(\frac{1}{12})m_{q}l_{3}{}^{2}{\left(\frac{{\dot{d}}_{ix}}{l_{3}}\right)}^{2} \end{array}$$

As limb *W* undergoes translational motions and contains limb *M*, the centre mobile platform, and the decoupler of ideal prismatic joints, the kinetic energies of limbs *W* in the *x* direction can be derived as

(40) $$T_{W}=\frac{1}{2}m_{W}\cdot {\dot{d}}_{ix}{}^{2}$$

The kinetic energy *T_O_* of the centre mobile platform can be calculated by

(41) $$T_{O}=\frac{1}{2}m_{o}\cdot {\dot{d}}_{iy}{}^{2}$$

where *m_o_* is the mass of the motion stage.

Then, the kinetic energy of the whole mechanism can be obtained as follows

(42) $$\begin{array}{cc} T= & (\frac{2}{3}m_{t}+\frac{1}{2}m_{v}+\frac{2}{3}m_{p}+\frac{1}{2}m_{e}+\frac{1}{24}m_{q}+\frac{1}{2}m_{W}){\dot{d}}_{ix}{}^{2}+(\frac{2}{3}m_{t}+\frac{1}{2}m_{v}+\frac{2}{3}m_{p}\\ & +\frac{1}{2}m_{o}+\frac{1}{2}m_{e}+\frac{1}{24}m_{q}){\dot{d}}_{iy}{}^{2} \end{array}$$

Next, the kinetic energy should be substituted into the Lagrange’s equation below,

(43) $$\frac{d}{dt}\frac{\partial T}{\partial {\dot{D}}_{I}}-\frac{\partial T}{\partial D_{I}}=Q_{j}$$

The dynamics equation of undamped free vibration of the compliant system can be derived as

(44) $$M\ddot{D}+KD=0$$

According to the characteristic equation, we have

(45) $$\left|K-M\lambda_{i}{}^{2}\right|=0$$

The natural frequency can be calculated by

(46) $$f=\frac{\lambda_{i}}{2\pi }=(\frac{1}{2\pi })\sqrt{\frac{K}{M}}$$

## 5. Workspace and Material Analysis

The relationship between the actual and nominal maximum output displacement of the PZT in the steady state can be expressed as

(47) $$D=\frac{k_{p}\Delta D-f_{pl}}{k_{p}+k_{s}}$$

where $k_{p}$ is the stiffness of the PZT, $k_{s}$ is the stiffness of spring load on the PZT, $f_{pl}$ is the preload on PZT, ΔD is the nominal stroke of the PZT without external load, and $k_{s}=k_{in}$ when the micro-motion stage has no external load.

### 5.1. Maximum Stress Subject to Rotation

If a flexure hinge bears a bending moment around its rotation axis, the maximum angular displacement $\theta^{\max}$ occurs when the maximum stress $\sigma^{\max}$ reaches the yield stress $\sigma^{y}$. The maximum stress usually appears at each outer surface of the thinnest part of the hinge. Therefore, the relationship between $\theta^{\max}$ and $\sigma^{\max}$ can be expressed as

(48) σmax=E(1+η)920η2f(η)θmax

where $\eta_{1}=w/l$ and $\eta_{2}=t/2r$ are dimensionless geometry factors and f(η) is a dimensionless compliance factor. The latter can be defined as

(49) f(η)=12η+η2[3+4η+2η2(1+η)(2η+η2)+6(1+η)(2η+η2)32tan−12+ηη]

According to the geometry of the micro-motion stage, the maximum angular deflection may occur on the hinge, which belongs to either bridge-type mechanism or level model. If the stage is actuated with a full stroke of the PZT, it could arrive at the maximum values [35]. Under such a case, we have $d_{1}=Ramp_{1}D$ and $d_{2}=RampD$.

Concerning the bridge-type mechanism, the flexure hinges rotation can be derived by

(50) $$\theta_{1}{}^{m}=\frac{d_{1}}{l_{a}}$$

For the lever model, the deformation of the flexure hinges can be expressed as

(51) $$\theta_{2}{}^{m}=\frac{d_{2}}{l_{b}}$$

According to Equations (48) and (51), the maximum stresses subject to the rotation of the flexures are obtained by

(52) σ1m=E(1+η1)920Ramp1Dη12f(η1)la, σ2m=E(1+η2)920RampDη22f(η2)lb

The following relationship must be satisfied to prevent material failure:

(53) $$S_{f}\sigma_{1}{}^{m}\leq \sigma_{y},\;S_{f}\sigma_{2}{}^{m}\leq \sigma_{y}$$

where $S_{f}\in \left(1,+\infty \right)$ is a specified safety factor. Substituting Equation (52) into (53) allows the derivation of the relationships,

(54) la≥E(1+η1)920Ramp1DSfη12f(η1)σy, lb≥E(1+η2)920RampDSfη22f(η2)σy

### 5.2. Maximum Tensile Stress Calculation

Under the axial load, the maximum tensile stress may occur on the thinnest portions of the flexure hinges constructing the bridge-type mechanism, which can be determined by

(55) $$\sigma_{3}{}^{m}=\frac{F_{in}}{S_{\min}}=\frac{K_{in}D}{2bw}$$

It should be noted that *bw* is multiplied by 2 because flexures *a* and *d* simultaneously bear the axial load. The maximum tensile stress should be limited to the allowable stress $\sigma_{y}$,

(56) $$\frac{K_{in}D}{bw}\leq \frac{2\sigma_{y}}{S_{f}}$$

In the other links of the stage, the maximum tensile stress in the thinnest part of flexure hinges can be expressed as

(57) $$\sigma_{4}^{m}=\frac{F_{1}}{S_{\min}}=\frac{u_{Ay}/d_{22}}{dt}$$

where $S_{\min}$ is the minimum cross-sectional area of the hinge. Then, it will lead to another constraint relationship for this design,

(58) $$\frac{u_{Ay}}{dt\cdot d_{22}}\leq \frac{\sigma_{y}}{S_{f}}$$

This is another guideline to avoid the risk of plastic failure in the size design of the stage.

## 6. Dimension Optimization

This chapter optimizes the dimensions of the 2D XY micro-motion stage to maximize the natural frequency, the determinant of the dynamic performance of the stage. Thus, the dimensions *r*, *t*, and *w* should be optimized. The analytical models on compliance and natural frequency show that the frequency has a nonlinear positive correlation with *t* and *w*, and a negative one with *r*. The increase of the frequency can significantly enhance the input stiffness and slightly reduce the quality. The parameters *t_a_*_1_, *t_a_*_2_ of the lever model and *l_a_* of the bridge-type mechanism are optimized to obtain the maximum amplification ratio. Further, parameter *l_b_* is optimized to achieve a compact structure. However, the optimal values of these parameters must be determined by computational method.

In this paper, grey wolves optimization (GWO) is introduced for dimensional optimization. Inspired by the leadership hierarchy and hunting mechanism of grey wolves. The applications of solving classical engineering design problems and the real problem in optical engineering have proven that the algorithm is suitable for challenging problems with unknown search spaces [36]. Compared with well-know heuristics genetic algorithms (GA), GWO is able highly competitive. GWO has a very high level of local optimal avoidance, which enhances the probability of finding proper approximations of the optimal weight and biases. Moreover, the accuracy of the optimal values for weights and biases is very high because of the high exploitation of GWO [37]. The optimization conditions are as follows:

(1) Objective: maximize the natural frequency (*f*)
(2) Related parameters: *r*, *t*, *w*, *t_a_*_1_, *t_a_*_2_, *l_a_*, and *l_b_*
(3) Constraints: parameters of right-circular flexure hinge: 0.05≤tr≤0.65; amplification ratio:$A_{m}\geq 6$; constraint Equations (54), (56), and (58); ranges of parameters: 0.3 mm≤t≤0.9 mm, 0.3 mm≤w≤0.9 mm, $17\;\mathrm{mm}\leq l_{a}\leq 22\;\mathrm{mm}$, $0.3\;\mathrm{mm}\leq t_{a1}\leq 0.9\;\mathrm{mm}$, $0.3\;\mathrm{mm}\leq t_{a1}\leq 0.9\;\mathrm{mm}$, $5\;\mathrm{mm}\leq l_{b}\leq 10\;\mathrm{mm}$, and 2 mm≤r≤4 mm.

The constraints are determined by the following factors: The value of *t*/*r* is limited to ensure the accuracy of the selected compliance factors *C_i_*. To prevent plastic failure of the material, the constraint equations of the stress analysis must be satisfied, and the safety factor *S_f_* is set to 1.5. In addition, the thinnest part of the flexible hinge should not be smaller than 0.3 mm; otherwise, the wire electro-discharge machining (WEDM) technique of the micro-motion stage cannot ensure the tolerance of 0.01 mm. Under lever bending and hinge stretching, the displacement loss may produce a sub-optimal lever amplification ratio in the optimization of lever dimension.

**Figure 16:**
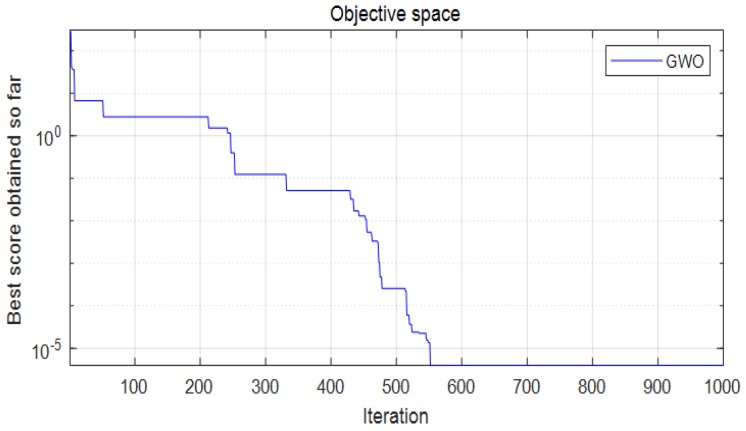
Convergence process of the grey wolves optimization (GWO).

The maximum number of iterations is set to 552. The convergence process of the GWO is presented in Figure 16. The optimal results are as follows: *t* = *w* = *t_a_*_1_ = 0.6 mm, *t_a_*_2_ = 0.4 mm, *l_a_* = 20.88 mm, *l_b_* = 8 mm, *r* = 3 mm (*t_a_*_1_, *t_a_*_2_ are the thickness of the right-angle hinge and the right-circular hinge, respectively, in the lever model). Based on these optimal dimensions, the natural frequency is computed as 234.885 Hz.

## 7. Performance Evaluation with Finite-Element Analysis

**Figure 17:**
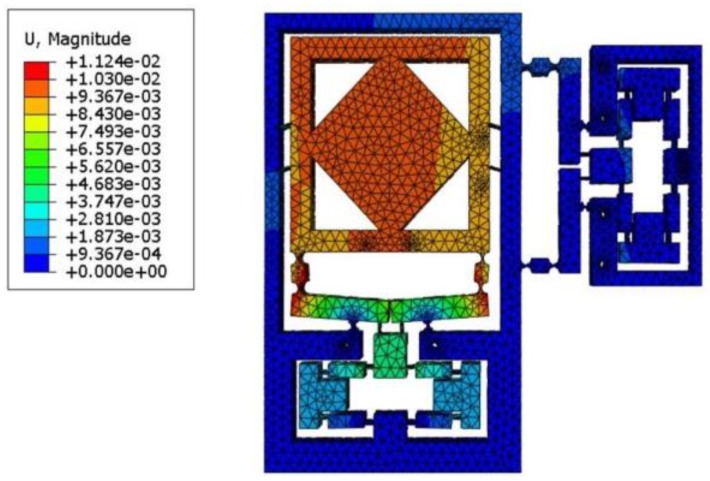
Deformation model XY micro-motion stage.

The output stiffness, input stiffness, amplification ratio, and natural frequency of the XY micro-motion stage were validated without damping on the finite element software ABAQUS/Explicit (Abaqus Inc., Palo Alto, CA, USA). The material used in the simulations was 6061 Al, for which the modulus of elasticity was 69,000 Mpa, possion’s ratio was 0.3, and density was 2700 kg/m^3^. Further, a 3.9 mm tetrahedral grid was chosen in mesh. The boundary conditions were set up after the geometric model meshing step and the input ends of the proposed micro-motion stage were applied to the load. The parameters of the model were described in Table 1. Sensitivity and convergence tests were performed at points B and C, respectively, as shown in Figure 15. During the EARP process, the hinges suffer from reciprocating tensile deformation, resulting in internal stress. Accordingly, internal stress on the compliance structure was tested in this paper. Specifically, the input load and output motion of the XY micro-motion stage were obtained as a given displacement on the input end of bridge-type mechanism; then, the input stiffness and amplification ratio of the flexure-based stage were determined based on the input load and output motion (Figure 17). Furthermore, the output stiffness can be evaluated by exerting an external force onto the stage.

**Figure 18:**
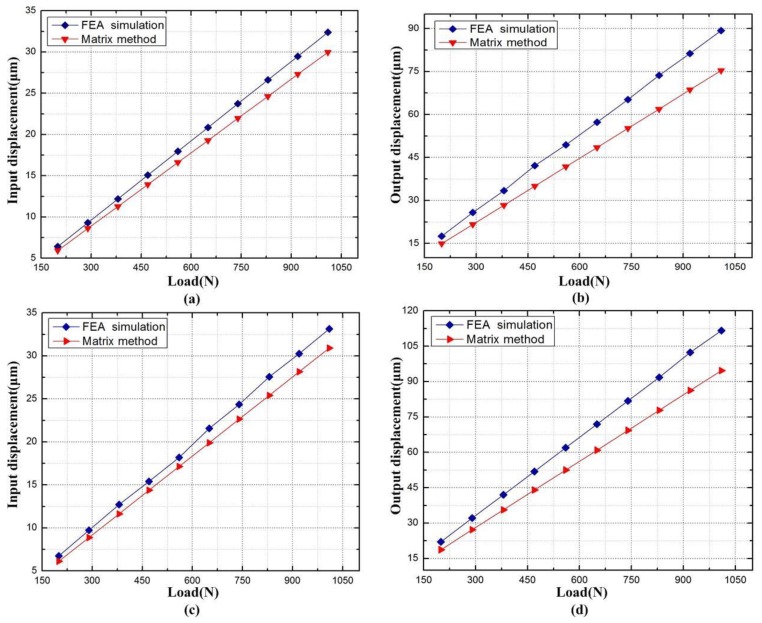
The force-displacement curves of XY micro-motion stage. (**a**) Input displacement in the *y* direction; (**b**) Output displacement in the *y* direction; (**c**) Input displacement in the *x* direction; (**d**) Output displacement in the *x* direction.

The force-displacement curves of XY micro-motion stage are presented in Figure 18. It can be seen from Figure 18a that the driving point displacement in the *x* direction increased linearly with the corresponding input load, when the stage was driven in *x* and *y* directions, respectively. The finite-element analysis shows that the input stiffness was 30.57 N/μm, 6.9% smaller than the theoretical value obtained by compliance matrix method. As shown in Figure 18c, the stiffness (31.2 N/μm) obtained by finite-element analysis was 7.4% smaller than that the theoretical value (33.71 N/μm) acquired by compliance matrix method. The output stiffness was tested by applying an external force on the flexure-based stage, and the resulting force-displacement curves are shown in Figure 18b,d.

In the *x* direction, the output stiffness was 9.02 N/μm, 18.3% smaller than the theoretical value 10.67 N/μm. In the y direction, the output stiffness was 11.31 N/μm, 18.6% smaller than the theoretical value 13.41 N/μm. The deviation mainly comes from the accuracy of the compliance factors equations and the hypothesized rigidity of these links in the matrix model.

**Figure 19:**
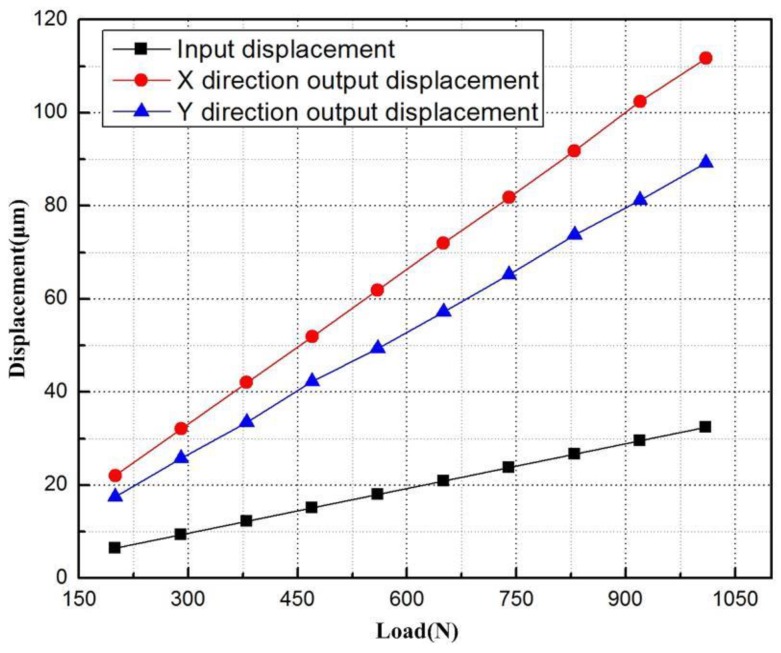
Input-output displacement relationship of the flexure-based stage.

Next, the finite-element analysis software was employed to measure the output displacements of the points on the sensor brackets. Similarly, the input displacements can be obtained by measuring the displacement of the input end in the bridge-type mechanism. The input-output displacement relationship of the flexure-based stage is displayed in Figure 19. It can be seen that the displacement amplification ratio was around 6.78 along the *x* axis, 11% smaller than the theoretical value of 7.55; that along the *y* axis was around 5.53, 6.5% greater than the theoretical value of 5.89. The differences are attributable to the fact that the deflections of the lever model reduced the displacement of the flexure-based stage. The deviation about displacement ratio between the *x* and *y* directions resulted from the displacement loss of the flexure hinges 7, 8, 9, and 10.

**Figure 20:**
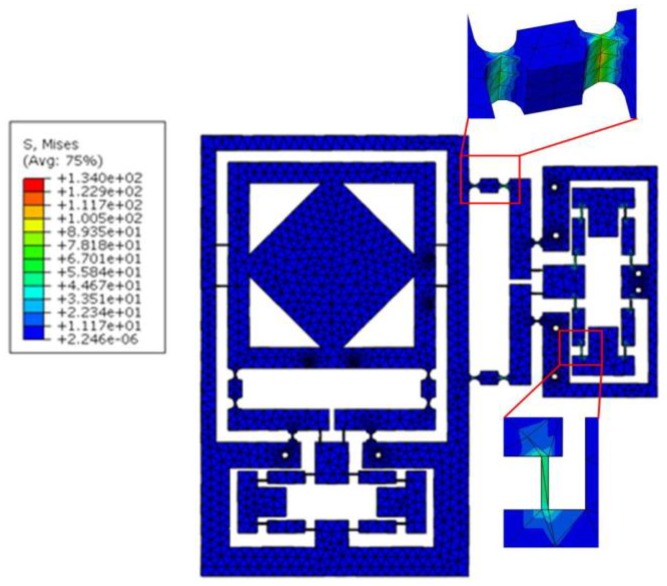
Stress test of the flexure-based stage.

The maximum stress of the thinnest hinge must be lower than the yield stress of the material (152 MPa). This is to prevent the hinges from failure in the production and application processes. To test the stress on the flexure-based stage, an input force $F_{I}={\left[1010,1010\right]}^{T}$ was applied at the input end so that the analysis results indicated the developed XY micro-motion stage possesses the potential to achieve the maximum workspace around 112 μm×89 μm, while the maximum von Mises stress are 62.09 MPa and 102.98 MPa, occurring in the right-angle hinge and right-circular hinge, respectively, without material failure as shown in Figure 20.

**Figure 21:**
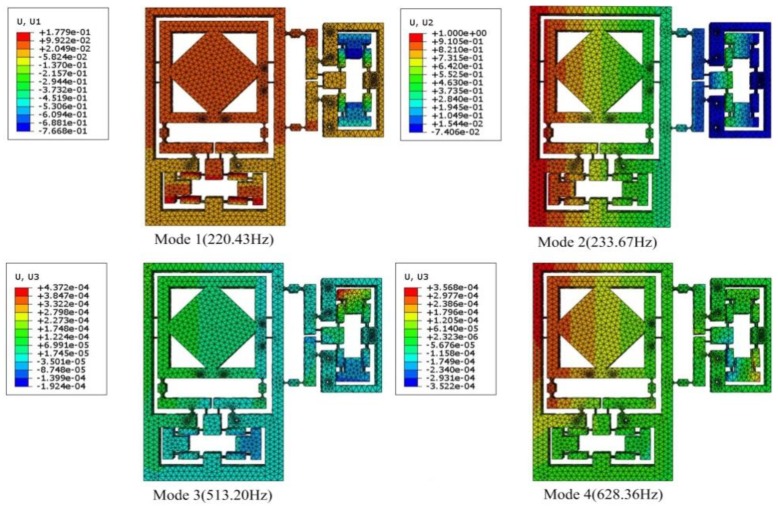
First four mode shapes of the flexure-based stage.

The first four mode shapes of the flexure-based stage (Figure 21) show that the two translational vibrations occurred at the first and the second modes with the frequencies 220.43 Hz and 233.67 Hz, respectively. Because of the structure symmetry, the first two modes had almost the same frequencies. The corresponding theoretical values obtained from Lagrange’s equation were both 239.7 Hz, 8.7% and 2.6% higher than the results of the finite-element analysis, respectively. The third and fourth modes had frequencies of 513.20 Hz and 628.36 Hz, leading to rotation and twisting, respectively. These two modes were neglected in analytical modelling, owing to the simulation difficulties.

## 8. Testing Experiments

**Figure 22:**
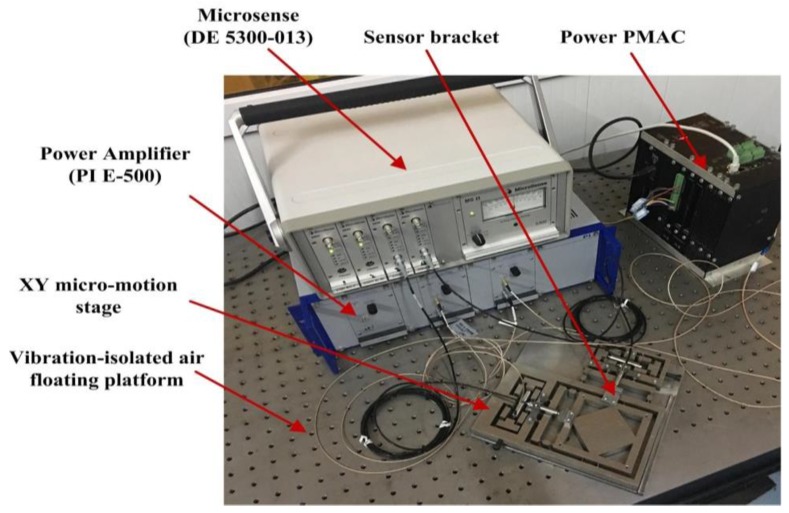
Schematic configuration of the experimental system.

Several offline tests were carried out to verify the performance of the XY micro-motion stage. The experimental setup is schematically shown in Figure 22. Two PZT actuators (40 vs. 12, Core Tomorrow Science Co., Ltd., Harbin, China), whose sizes are φ12 mm×51.5 mm and $K_{\mathrm{pzt}}=35\;N/\mu m$, respectively, were embedded into the drive structures to achieve impact structure sizes. A power amplifier (PI, E-500) was utilized to amplify the excitation signals, which were generated by the power PMAC controller. A four-channel capacitive displacement sensor (Micro-sense DE 5300-013) was chosen for dynamic position measurements. To reduce external disturbances on the sensing and measurement system, a vibration-isolated air-floating platform was used to mount the XY micro-motion stage.

### 8.1. Sine Sweep Response

**Figure 23:**
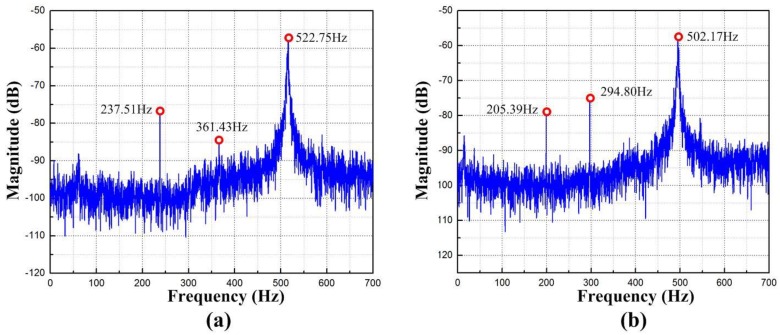
Dynamic responses along (**a**) *y* axis and (**b**) *x* axis.

The swept excitation method is chosen to study the dynamic characteristic as it is an expedient method to use. A variable frequency sine sweep signal was applied to the PZT actuators for each axis, and the displacement response was recorded and analyzed by fast Fourier transform (FFT). The measured result along *y* and *x* axes are shown in Figure 23a,b. It can be obtained from Figure 23a that the first three natural frequencies were 237.51 Hz, 361.43 Hz, and 522.75 Hz, respectively. Figure 23b shows the first three natural frequencies were 205.39 Hz, 294.80 Hz, and 502.17 Hz, which were coincident with the first three natural frequencies measured along the *x* axis as well. The marginal difference may be caused by manufacturing errors and additional mass. According to a higher increase in the equivalent masses compared with the increase in stiffness, the frequencies obtained are relatively smaller. The working bandwidth of the machining system is limited by the lowest first natural frequency. If the contacts between PZT actuators and input ends perfectly, a micro-motion in a high frequency range can be achieved [38]. However, the resonance frequencies satisfy the requirements of precision polishing at medium or low roll speed.

### 8.2. Stroke and Resolution Test

**Figure 24:**
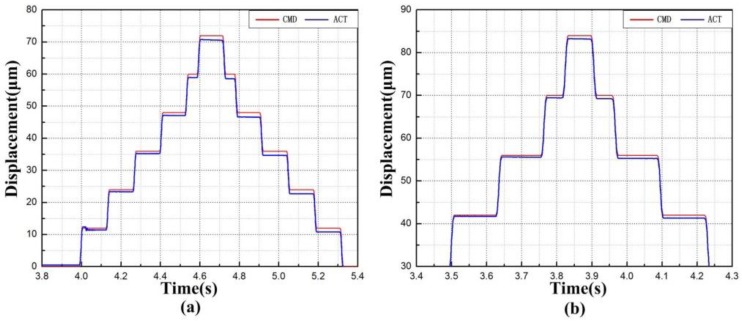
Motion stroke of (**a**) *x* axis and (**b**) *y* axis. CMD—command displacement; ACT—actual displacement.

The surface roughness, a key to precision polishing, is positively correlated with the stroke of XY micro-motion stage. In light of this, a stair control voltage with maximum displacement of 72 µm was given to the *x* direction actuator. Similarly, the maximum displacement of 84 µm was adopted as the driving signal in the *y* direction. Figure 24 records the displacements in the *x* and *y* directions. In the figure, CMD means command displacement, while ACT refers to the actual displacement. It can be seen that the stroke was 71 µm and 83 µm along *x* and *y* axes, respectively. Moreover, Figure 24b shows that the compliant mechanism cannot track the maximum input command immediately, which demonstrates the tested compliant mechanism is working in the maximum limitation of displacement output. There is a huge difference between simulated and actual displacements along the *x* axis. A possible reason lies in the friction between the flexure-based stage and fixed base plate, which is generated by manufacturing accuracy and assembly errors. If the contact surface is sufficiently smooth, the actual displacement could approximate the simulated results.

**Figure 25:**
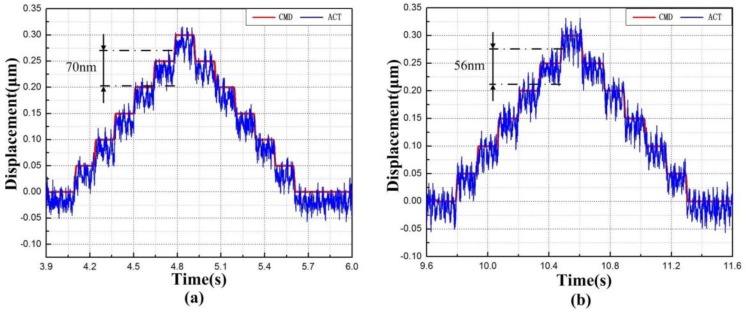
Resolution along the (**a**) *x* axis and (**b**) *y* axis.

The resolution is a major design criterion for the flexure-based stage. In theory, the piezo-electric-driven flexure-based stage can achieve a high resolution. To identify the resolution of the flexure-based stage, a stair-step command signal was generated from a digital computer, which was provided to the PEAs to drive flexure stage. In each step, chattering was observed mainly because of the inherent noise effect of the capacitance sensor. The responses in Figure 25a,b show that the flexure-based stage achieved the resolution of 70 nm and 56 nm along the *x* and *y* axes, respectively. If the performance of the capacitance sensor could be improved and external disturbance is reduced, a higher output resolution (~50 nm) can be obtained [39].

### 8.3. Step Response and Sine Response Test

**Figure 26:**
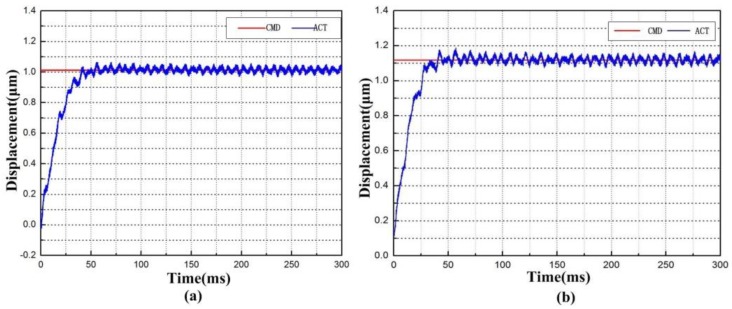
Step responses of the apparatus in (**a**) the *x* direction and (**b**) the *y* direction.

The step and sine responses along the two directions were examined to inspect the XY micro-motion stage performance. A typical proportional-integral-derivative (PID) controller was implemented to position the flexure-based stage. According to the results in Figure 26, the rise times of the *x* axis and y axis motions were approximately 29 ms and 24 ms, respectively. Moreover, very small and steady errors and overshoots were observed thanks to the feedback control during the positioning process.

To sum up, all the test results show that the proposed EARP system can follow external commands with fast response speed and high accuracy, and thus can be applied to precision polishing.

## 9. Polishing Experiment

### 9.1. Experiment Setup

**Figure 27:**
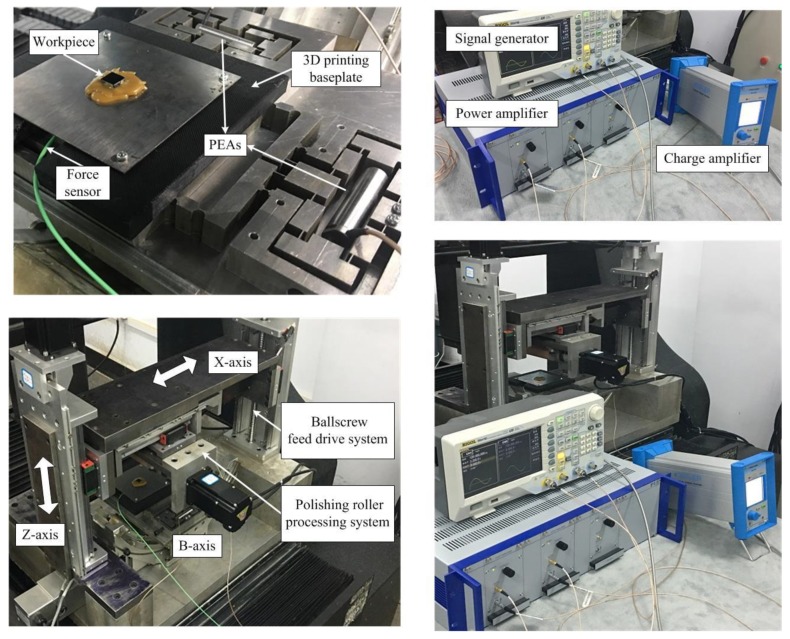
Polishing experimental setup.

The polishing experiments were performed on the independently developed vibration-assisted roll-type polishing machine. Figure 27 shows photographs of the machine. The polishing roll is mounted on an XZ table by linear guides. The micro-motion stage is mounted on a B-axis tilting table and the workpiece is mounted on the flexure stage with bonding wax. The experiment setup consists of the machines, the micro-motion stage, the charge amplifer (Type 5018, Kistler, Winterthur, Switzerland), the force sensor (Type 9211B, Kistler), the PEAs (40 vs. 12, Core Tomorrow Science Co., Ltd., Harbin, China), signal generator (DG4162, Rigol, Beijing, China), power amplifier (PI, E-500), and so on. The control signals provided by the signal generator were amplified using power amplifier and were then sent to drive the piezo-electric stacks. To achieve accurate polishing, the force sensor was applied to detect abrasive-workpiece engagement and the charge amplifier was used to measure the polishing forces.

**Figure 28:**
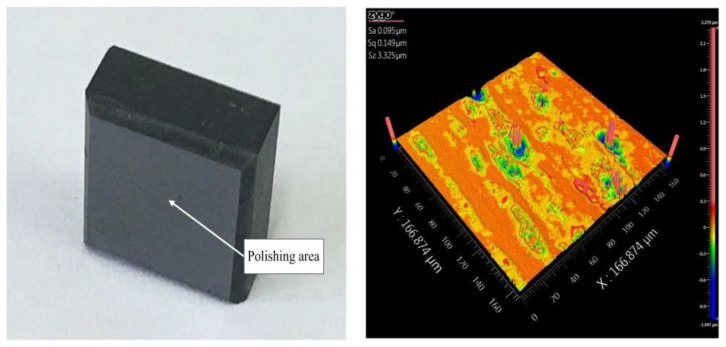
Silicon carbide ceramic workpiece.

The specific experimental parameters were as follows: roll speed: 240 r/min; frequency (*f*_1_, *f*_2_): 120 Hz, 120 Hz; amplitude (A_1_, A_2_): 5 μm, 6 μm; input voltage (V_1_, V_2_): 4 V, 8 V; phase difference: $90^{\circ }$. The workpiece material was ground prior to polishing of SiC ceramic, as shown in Figure 28. The white-light interferometer (ZygoNewview, Middlefield, CT, USA) was employed to capture the topography features.

### 9.2. Results and Discussions

**Figure 29:**
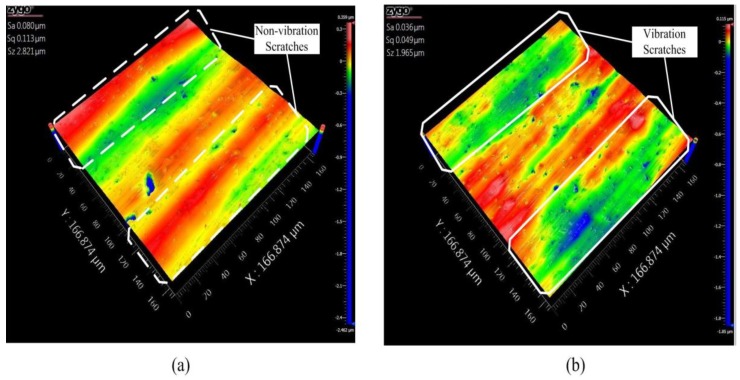
Workpiece surface morphology (**a**) non-vibration polishing and (**b**) vibration polishing.

The workpiece surface morphology with non-vibration and vibration polishing are given in Figure 29. Because of the unstable control of polishing slurry, some marks can be observed at the workpiece with both methods. Figure 29a shows the surface roughness was improved from 95 nm Sa to 80 nm Sa and 504 nm Sz reduced with form deviation by non-vibration polishing. The polishing scratches can apparently be observed in the area. As shown in Figure 29b, the scratches were increased compared with the former as a result of more abrasives involved in polishing (44 nm Sa), and 856 nm Sz smaller than non-vibration by applying vibration. The performance of the vibration assisted roll-type polishing was evaluated by experiment, indicating that the developed method could effectively improve the surface quality.

## 10. Conclusions

In this paper, the aim was the generation of a high-quality surface based on the linear contact material removal mechanism. The theoretical model for the micro-motion stage’s statics, compliance, and dynamic properties were given and validated by the GWO, followed by prototype fabrication. A closed-loop test and polishing experiment were determined to meet the critical requirements for precision finishing. Compared with existing mechanisms, dedicated to polishing, the advantages of the proposed EARP system are listed as follows:

(1) It is capable to deliver large 2D vibration amplitudes, while maintaining favourable resolution. Experimental tests performed show that the vibration strokes in the *x*- and *y*-directions can reach 71 μm and 83 μm, respectively, while the resolution at points B and C of the micro-motion stage are 70 nm and 56 nm, respctively.
(2) Because of the high stiffness of the proposed flexure-based stage, natural frequencies that meet the requirements are also achieved, which were examined to be 205.39 Hz and 237.51 Hz in the *x*- and *y*-directions, respectively.
(3) Compared with the non-vibration roll-type polishing system, 44 nm Sa and 856 nm Sz are improved by proposed EARP system used independently developed polishing machine. Accordingly, the micro-motion stage could increase the number of abrasive particles involved in polishing.

## Figures and Tables

**Table 1:** Main parameters of the flexure-based stage.

***r***	***b***	***t***	***w***	***t_a_*_1_**	***t_a_*_2_**
3 mm	15 mm	0.6 mm	0.6 mm	0.6 mm	0.4 mm
***l_a_***	***l_b_***	***E***	***σ***	***μ***	***ρ***
20.88 mm	8 mm	69,000 MPa	228 MPa	0.3	2700 kg/m^3^
